# Functional Connectivity Patterns Associated with Inflammation in Psychosis; Results From the UK Biobank Database

**DOI:** 10.1192/bjo.2025.10102

**Published:** 2025-06-20

**Authors:** Edward Palmer, Rachel Upthegrove, Jack Rogers

**Affiliations:** ^1^University of Birmingham, Birmingham, United Kingdom; ^2^Birmingham and Solihull Mental Health foundation Trust, Birmingham, United Kingdom; ^3^University of Oxford, Oxford, United Kingdom; ^4^University of Birmingham, Birmingham, United Kingdom

## Abstract

**Aims:** Recent evidence suggests that inflammation and immune dysregulation play a role in mental health disorders, including psychosis. Research has identified grey matter volume changes, however, the relationship between inflammation and functional connectivity remains underexplored. This study investigates the impact of CRP levels on functional connectivity in psychosis.

**Methods:** This study used data from the UK Biobank (project 92051), an open-access resource with demographic, clinical, and neuroimaging data for over 500,000 individuals aged 40–69. We identified 91 participants with a psychotic disorder and matched 91 healthy controls (HCs). Neuroimaging data were analysed using the CONN toolbox in MATLAB, focusing on ROI-to-ROI functional connectivity, calculating Fisher z-transformed Pearson correlations, and using general linear models (GLM) for statistical comparisons. Multiple comparisons were corrected with False Discovery Rate (FDR). The interaction between functional connectivity and inflammation was examined using CRP levels as a continuous variable. Group-level analyses employed multivariate parametric statistics with random-effects modelling and Gaussian Random Field theory, with significance thresholds set at p<0.001 (voxel) and p-FDR<0.05 (cluster).

**Results:** ROI-to-ROI analysis revealed significant connectivity changes between psychosis cases and HCs, modulated by CRP. After adjusting for whole-brain connectivity, a cluster of hypoconnectivity was found between temporal regions and the language network (F(4, 179)=4.55, p-FDR=0.04). A second hypoconnectivity cluster involved the bilateral insular cortex and the sensory-motor cortex (F(4, 179)=4.49, p-FDR=0.04).

A third cluster, mostly showing hypoconnectivity, was found between the bilateral cerebellum and the right temporal gyrus (F(4, 179)=5.23, p-FDR=0.04), with hyperconnectivity specifically between the anterior middle temporal gyrus and cerebellum. These regions, especially the left superior temporal gyrus, left insula, and cerebellum, have previously been linked to psychosis and negative symptoms.

At the network level, hypoconnectivity was observed within the salience network, including between the rostral prefrontal cortex, insula, supramarginal gyrus, and anterior cingulate cortex (p-FDR=0.03–0.05). Hyperconnectivity was found between the salience network and default mode network (F(4, 179)=3.52, p-FDR=0.03). This pattern of hypoconnectivity within networks but increased cross-network connectivity mirrors findings in first-episode psychosis with auditory hallucinations.

**Conclusion:** This study highlights significant CRP-modulated functional connectivity changes in psychosis, particularly hypoconnectivity within the temporal, insular, and motor regions, as well as the salience network. Hyperconnectivity between the salience and default mode networks was also observed. These findings suggest inflammation’s role in neural dysregulation.

